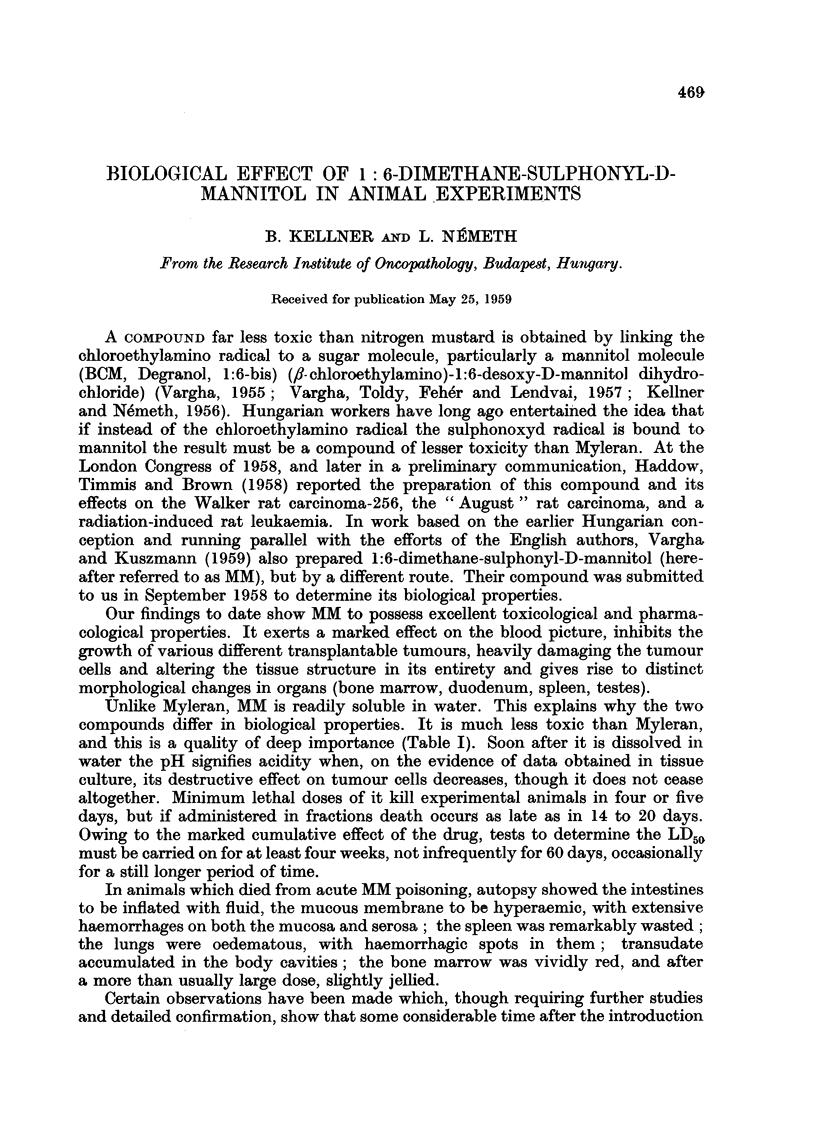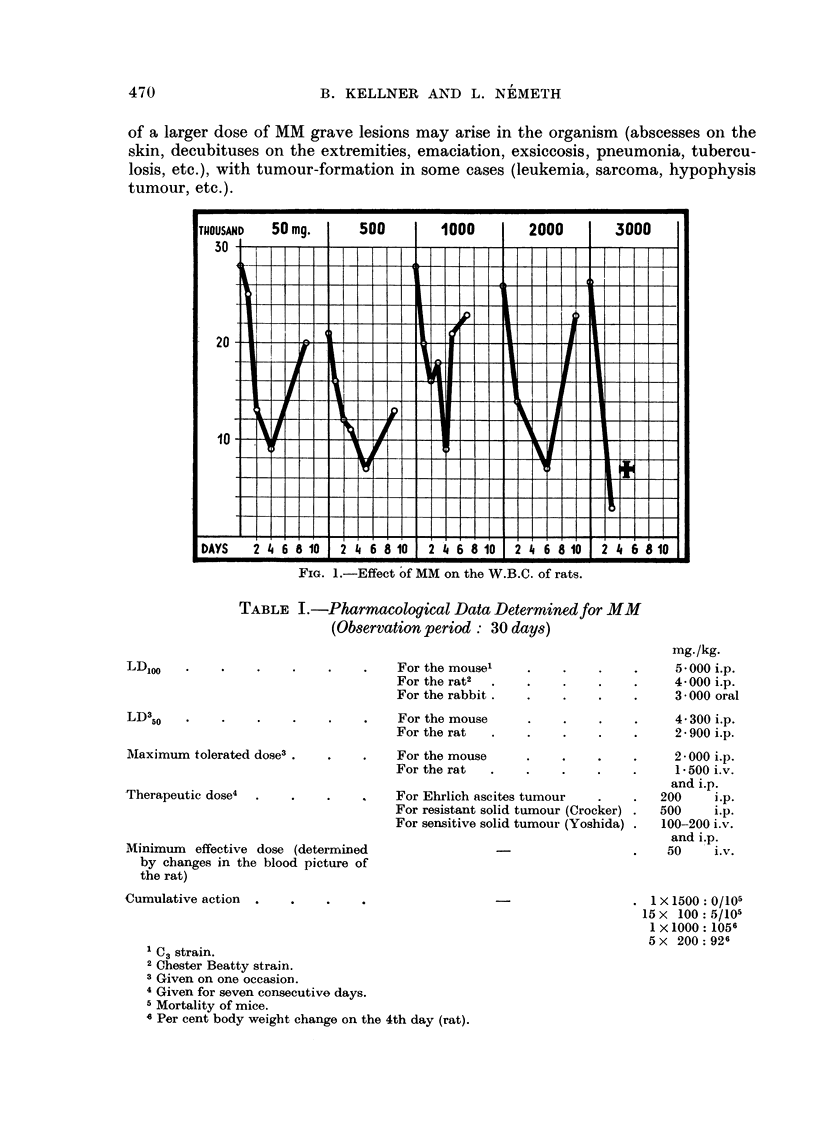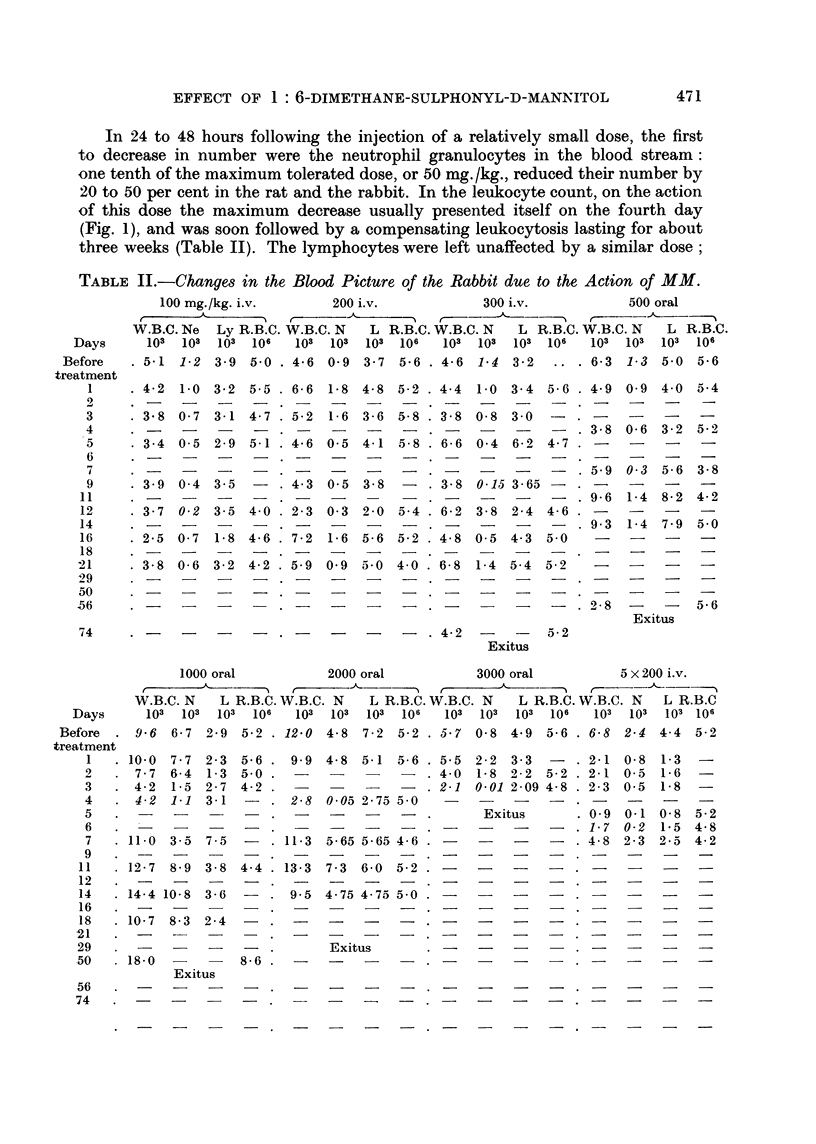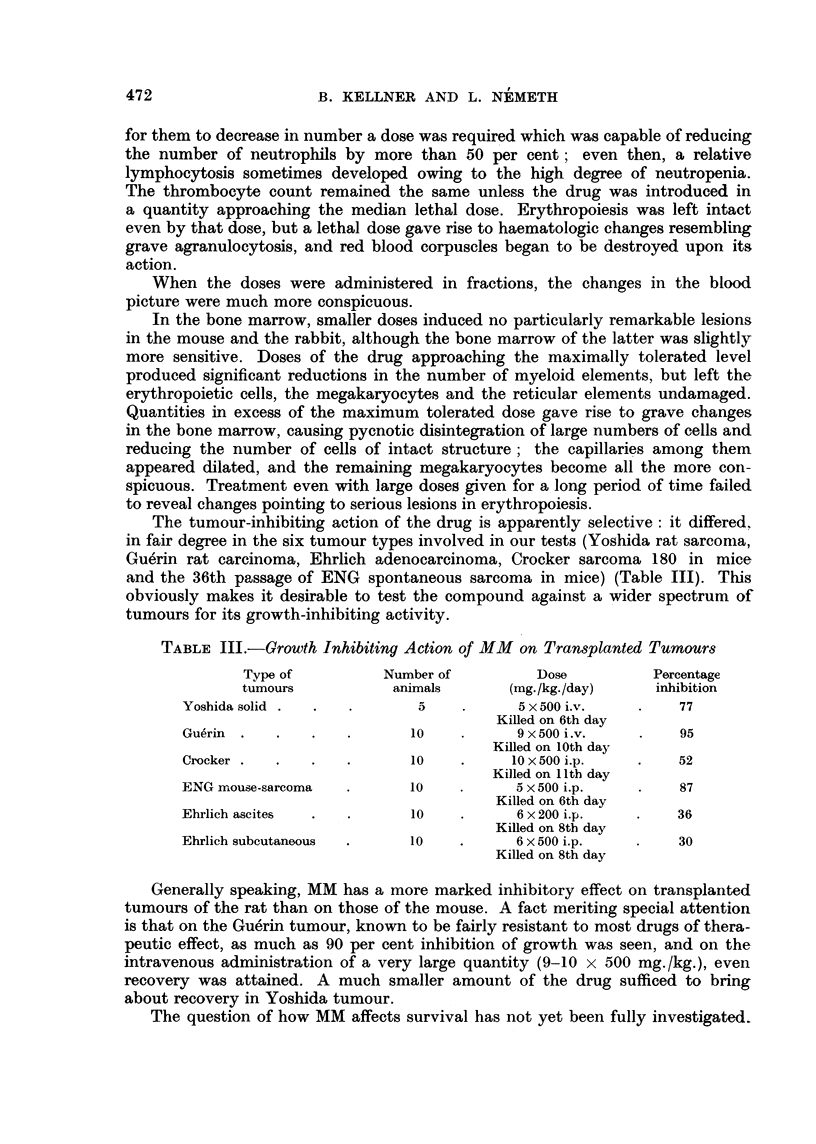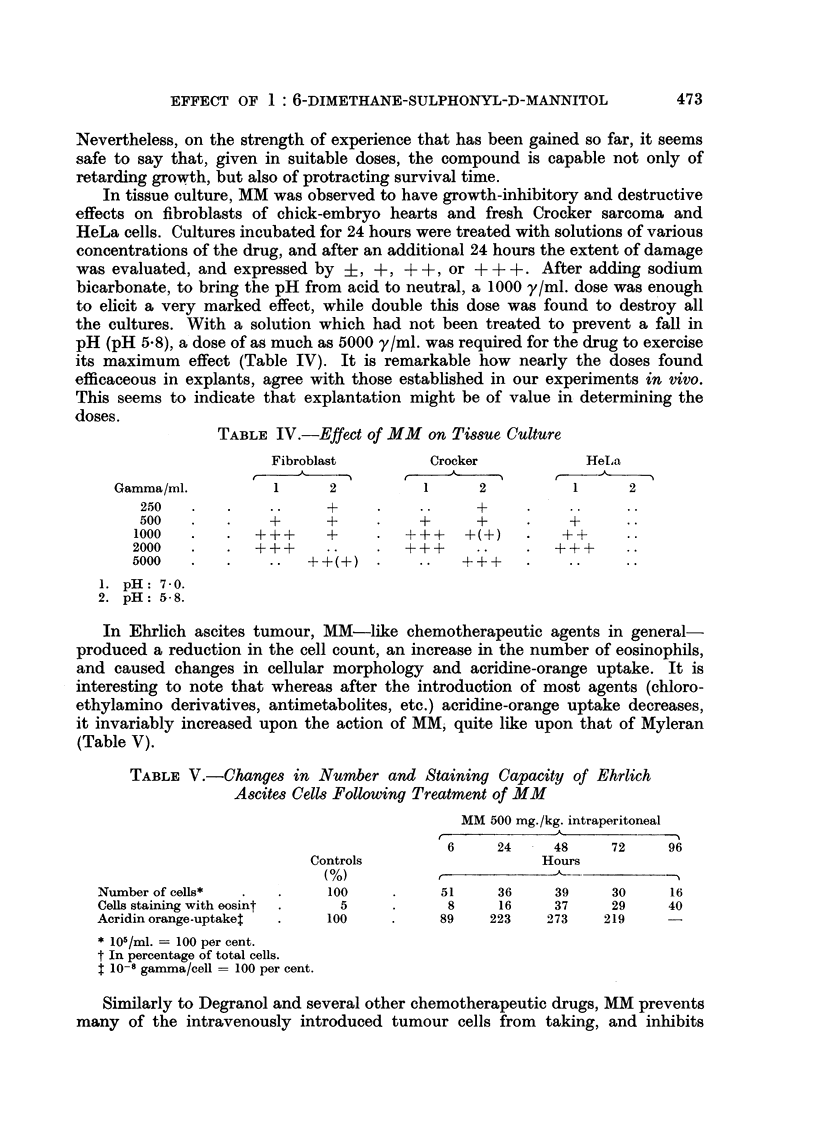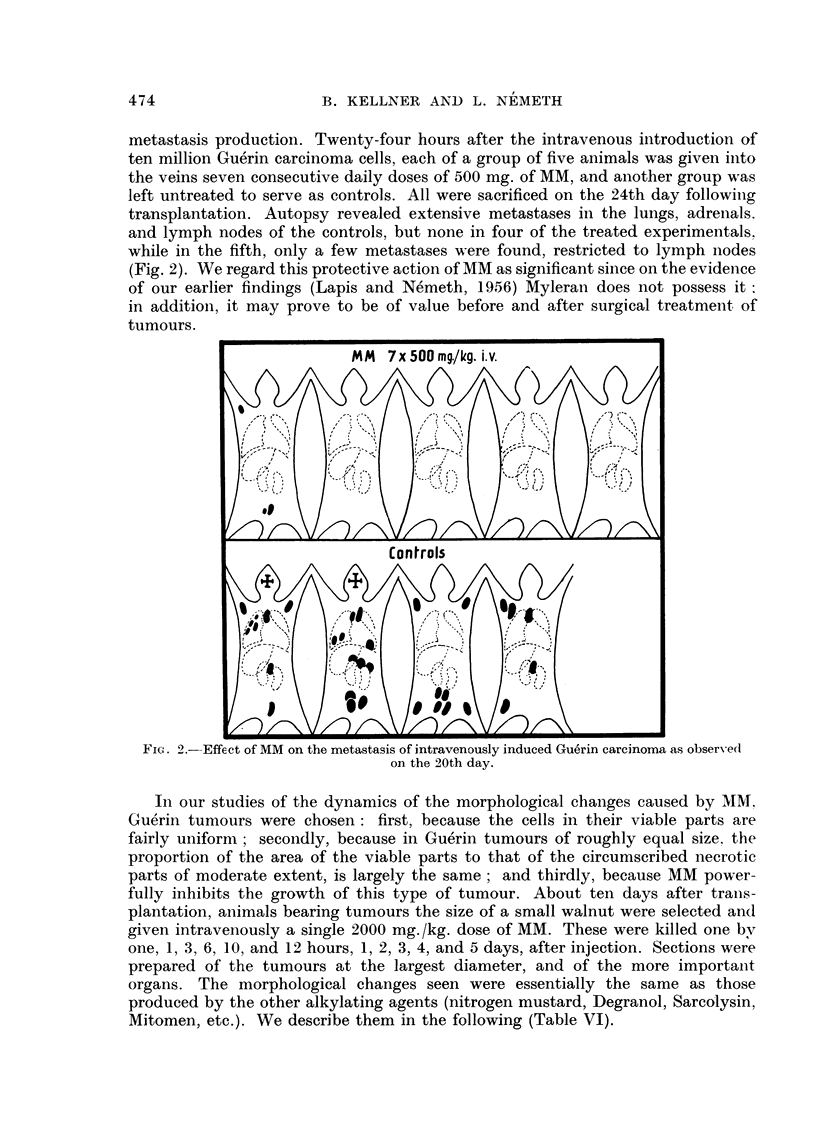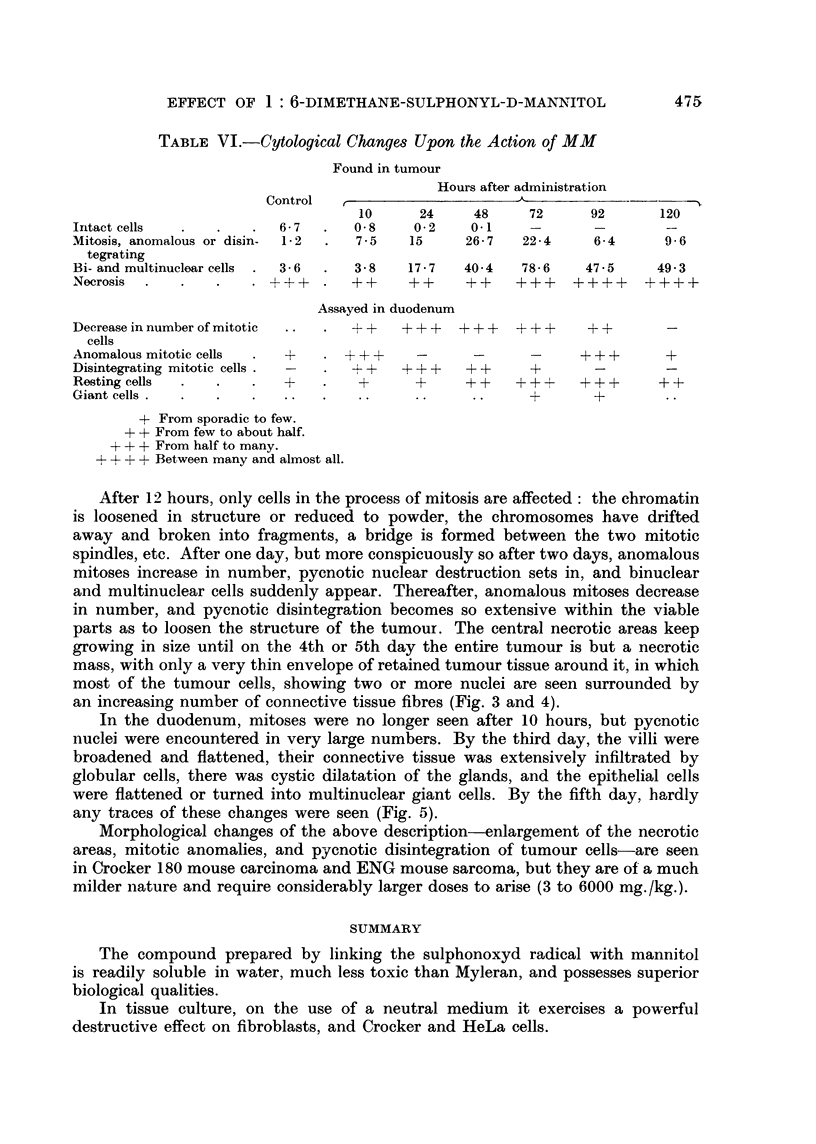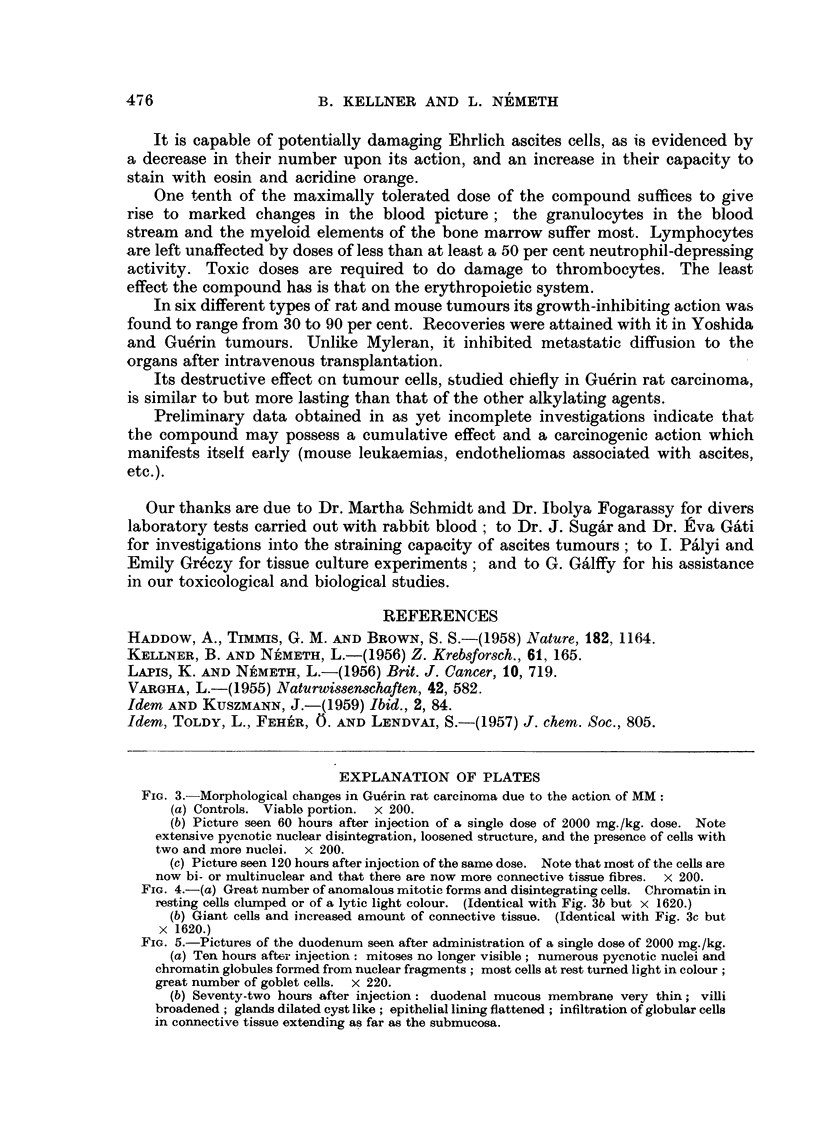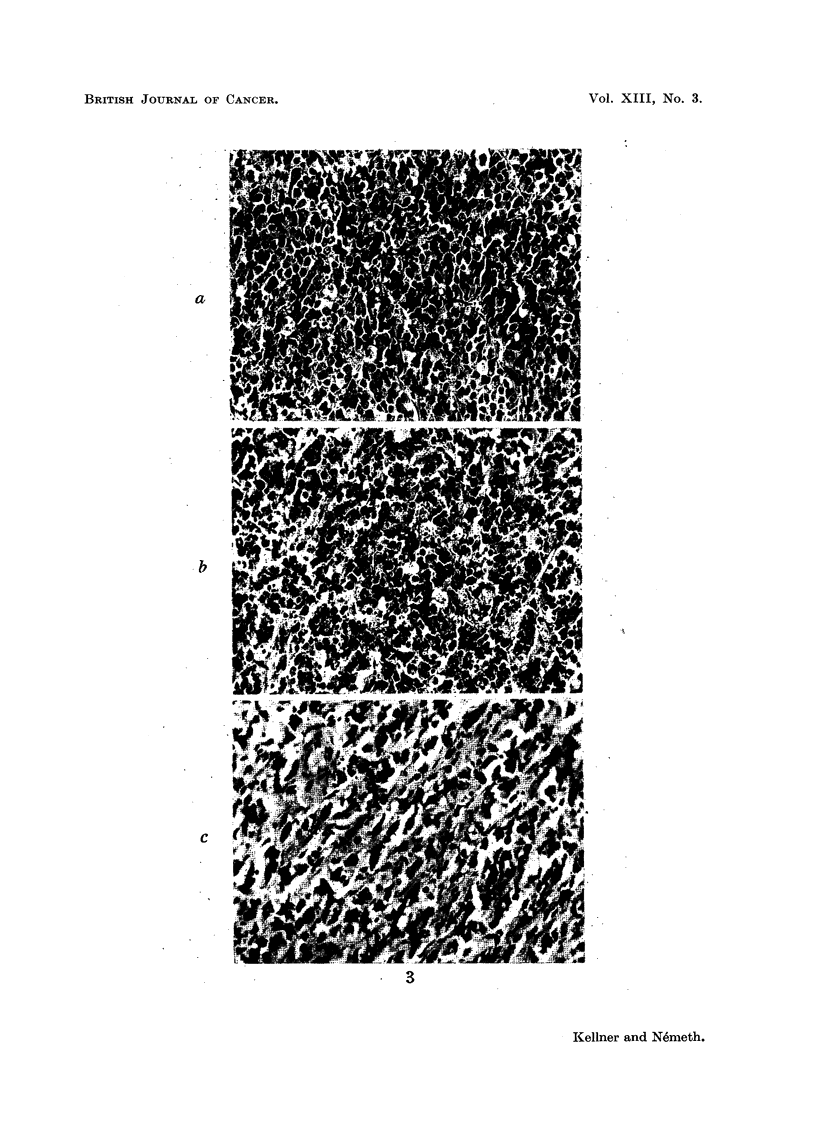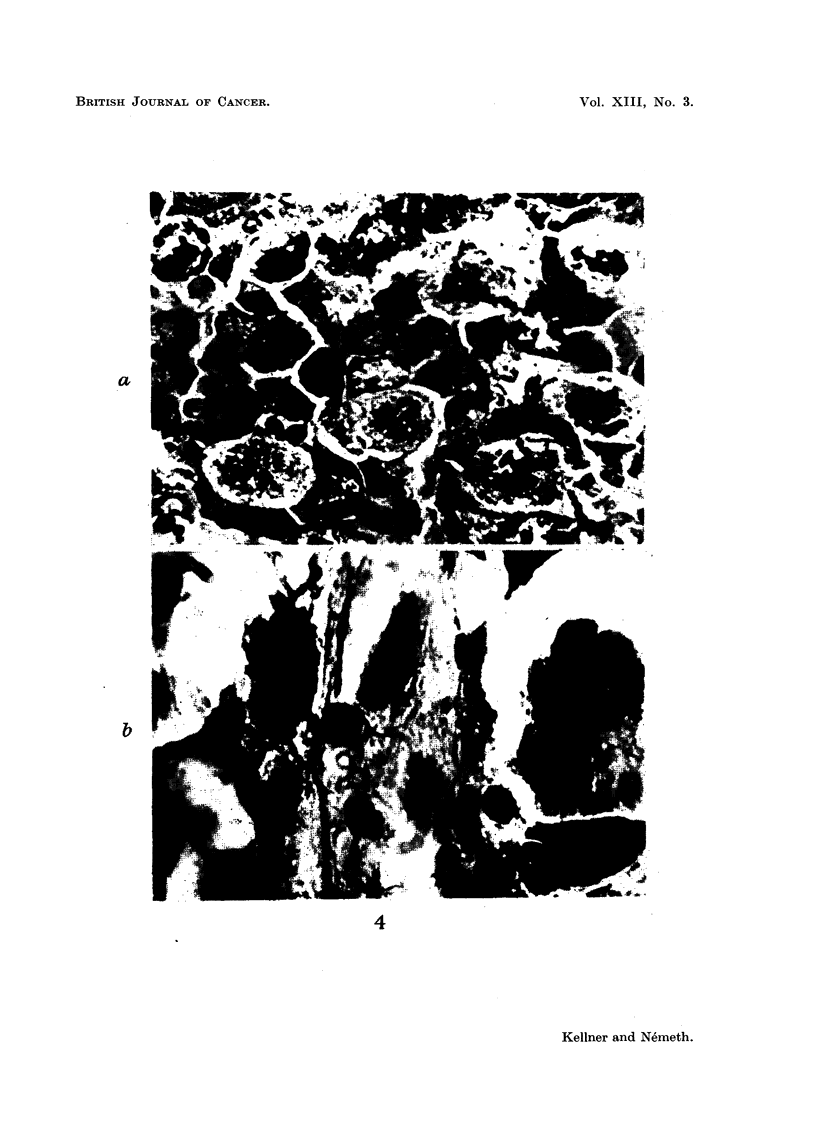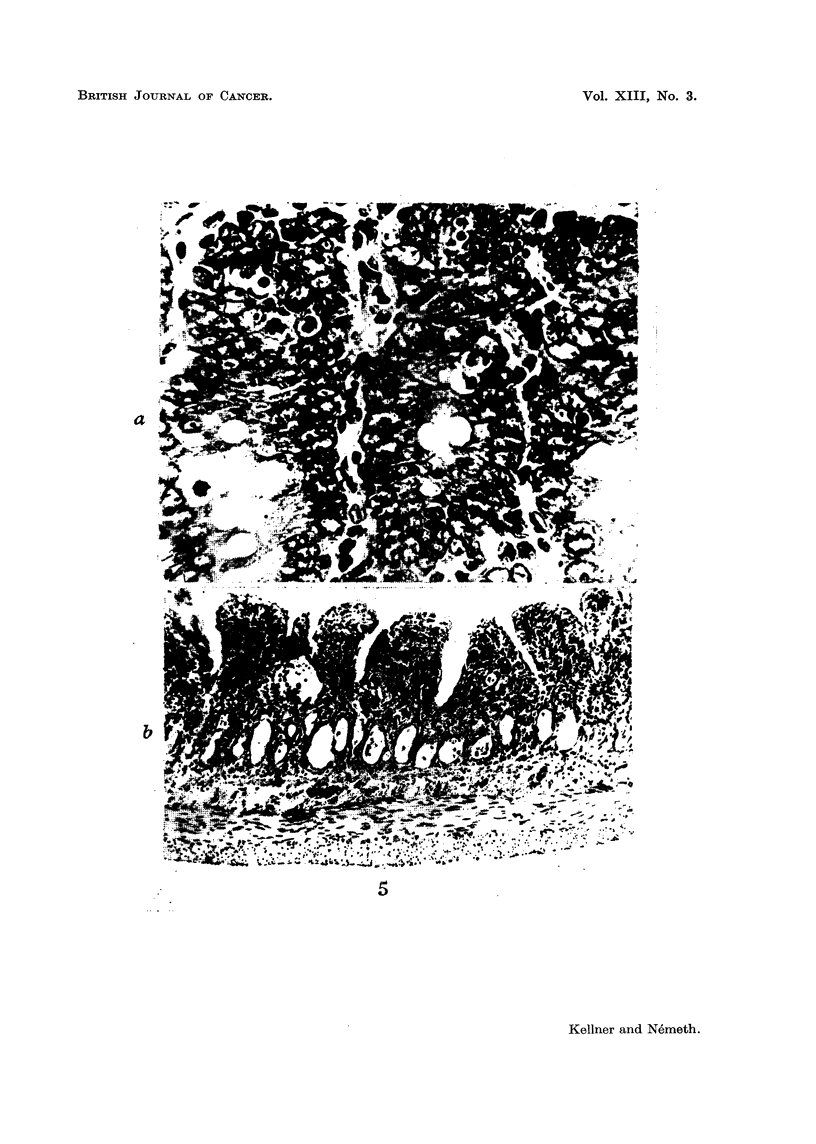# Biological Effect of 1:6-Dimethane-Sulphony-D-Mannitol in Animal Experiments

**DOI:** 10.1038/bjc.1959.52

**Published:** 1959-09

**Authors:** B. Kellner, L. Németh

## Abstract

**Images:**


### BIOLOGICAL EFFECT OF 1 : 6-DIMETHANE-SULPHONYL-D-MANNITOL IN ANIMAL EXPERIMENTS

B. KELLNER AND L. NÉMETH

*From the Research Institute of Oncopathology, Budapest, Hungary.*

Received for publication May 25, 1959

A COMPOUND far less toxic than nitrogen mustard is obtained by linking the chloroethylamino radical to a sugar molecule, particularly a mannitol molecule (BCM, Degranol, 1:6-bis) ($\beta$-chloroethylamino)-1:6-desoxy-D-mannitol dihydrochloride) (Vargha, 1955 ; Vargha, Toldy, Fehér and Lendvai, 1957 ; Kellner and Németh, 1956). Hungarian workers have long ago entertained the idea that if instead of the chloroethylamino radical the sulphonoxyd radical is bound to mannitol the result must be a compound of lesser toxicity than Myleran. At the London Congress of 1958, and later in a preliminary communication, Haddow, Timmis and Brown (1958) reported the preparation of this compound and its effects on the Walker rat carcinoma-256, the " August " rat carcinoma, and a radiation-induced rat leukaemia. In work based on the earlier Hungarian conception and running parallel with the efforts of the English authors, Vargha and Kuszmann (1959) also prepared 1:6-dimethane-sulphonyl-D-mannitol (hereafter referred to as MM), but by a different route. Their compound was submitted to us in September 1958 to determine its biological properties.

Our findings to date show MM to possess excellent toxicological and pharmacological properties. It exerts a marked effect on the blood picture, inhibits the growth of various different transplantable tumours, heavily damaging the tumour cells and altering the tissue structure in its entirety and gives rise to distinct morphological changes in organs (bone marrow, duodenum, spleen, testes).

Unlike Myleran, MM is readily soluble in water. This explains why the two compounds differ in biological properties. It is much less toxic than Myleran, and this is a quality of deep importance (Table I). Soon after it is dissolved in water the pH signifies acidity when, on the evidence of data obtained in tissue culture, its destructive effect on tumour cells decreases, though it does not cease altogether. Minimum lethal doses of it kill experimental animals in four or five days, but if administered in fractions death occurs as late as in 14 to 20 days. Owing to the marked cumulative effect of the drug, tests to determine the $LD_{50}$ must be carried on for at least four weeks, not infrequently for 60 days, occasionally for a still longer period of time.

In animals which died from acute MM poisoning, autopsy showed the intestines to be inflated with fluid, the mucous membrane to be hyperaemic, with extensive haemorrhages on both the mucosa and serosa ; the spleen was remarkably wasted ; the lungs were oedematous, with haemorrhagic spots in them ; transudate accumulated in the body cavities ; the bone marrow was vividly red, and after a more than usually large dose, slightly jellied.

Certain observations have been made which, though requiring further studies and detailed confirmation, show that some considerable time after the introduction of a larger dose of MM grave lesions may arise in the organism (abscesses on the skin, decubituses on the extremities, emaciation, exsiccosis, pneumonia, tuberculosis, etc.), with tumour-formation in some cases (leukemia, sarcoma, hypophysis tumour, etc.).

FIG. 1.—Effect of MM on the W.B.C. of rats.

TABLE I.—*Pharmacological Data Determined for MM (Observation period : 30 days)*

| | | mg./kg. |
|---|---|---|
| $LD_{100}$ . . . . . . . | For the mouse[1] . . . . . | 5·000 i.p. |
| | For the rat[2] . . . . . . | 4·000 i.p. |
| | For the rabbit . . . . . . | 3·000 oral |
| $LD_{50}$[3] . . . . . . . | For the mouse . . . . . | 4·300 i.p. |
| | For the rat . . . . . . | 2·900 i.p. |
| Maximum tolerated dose[3] . . . | For the mouse . . . . . | 2·000 i.p. |
| | For the rat . . . . . . | 1·500 i.v. and i.p. |
| Therapeutic dose[4] . . . . . | For Ehrlich ascites tumour . . | 200 i.p. |
| | For resistant solid tumour (Crocker) . | 500 i.p. |
| | For sensitive solid tumour (Yoshida) . | 100–200 i.v. and i.p. |
| Minimum effective dose (determined by changes in the blood picture of the rat) | — . | 50 i.v. |
| Cumulative action . . . . . | — . | 1×1500 : 0/10[5]<br>15× 100 : 5/10[5]<br>1×1000 : 105[6]<br>5× 200 : 92[6] |

[1] $C_3$ strain.
[2] Chester Beatty strain.
[3] Given on one occasion.
[4] Given for seven consecutive days.
[5] Mortality of mice.
[6] Per cent body weight change on the 4th day (rat).

In 24 to 48 hours following the injection of a relatively small dose, the first to decrease in number were the neutrophil granulocytes in the blood stream : one tenth of the maximum tolerated dose, or 50 mg./kg., reduced their number by 20 to 50 per cent in the rat and the rabbit. In the leukocyte count, on the action of this dose the maximum decrease usually presented itself on the fourth day (Fig. 1), and was soon followed by a compensating leukocytosis lasting for about three weeks (Table II). The lymphocytes were left unaffected by a similar dose ;

TABLE II.—*Changes in the Blood Picture of the Rabbit due to the Action of MM.*

| | 100 mg./kg. i.v. | | | | 200 i.v. | | | | 300 i.v. | | | | 500 oral | | | |
|---|---|---|---|---|---|---|---|---|---|---|---|---|---|---|---|---|
| Days | W.B.C. $10^3$ | Ne $10^3$ | Ly $10^3$ | R.B.C. $10^6$ | W.B.C. $10^3$ | N $10^3$ | L $10^3$ | R.B.C. $10^6$ | W.B.C. $10^3$ | N $10^3$ | L $10^3$ | R.B.C. $10^6$ | W.B.C. $10^3$ | N $10^3$ | L $10^3$ | R.B.C. $10^6$ |
| Before treatment | 5·1 | *1·2* | 3·9 | 5·0 | 4·6 | 0·9 | 3·7 | 5·6 | 4·6 | *1·4* | 3·2 | .. | 6·3 | *1·3* | 5·0 | 5·6 |
| 1 | 4·2 | 1·0 | 3·2 | 5·5 | 6·6 | 1·8 | 4·8 | 5·2 | 4·4 | 1·0 | 3·4 | 5·6 | 4·9 | 0·9 | 4·0 | 5·4 |
| 2 | — | — | — | — | — | — | — | — | — | — | — | — | — | — | — | — |
| 3 | 3·8 | 0·7 | 3·1 | 4·7 | 5·2 | 1·6 | 3·6 | 5·8 | 3·8 | 0·8 | 3·0 | — | — | — | — | — |
| 4 | — | — | — | — | — | — | — | — | — | — | — | — | 3·8 | 0·6 | 3·2 | 5·2 |
| 5 | 3·4 | 0·5 | 2·9 | 5·1 | 4·6 | 0·5 | 4·1 | 5·8 | 6·6 | 0·4 | 6·2 | 4·7 | — | — | — | — |
| 6 | — | — | — | — | — | — | — | — | — | — | — | — | — | — | — | — |
| 7 | — | — | — | — | — | — | — | — | — | — | — | — | 5·9 | *0·3* | 5·6 | 3·8 |
| 9 | 3·9 | 0·4 | 3·5 | — | 4·3 | 0·5 | 3·8 | — | 3·8 | *0·15* | 3·65 | — | — | — | — | — |
| 11 | — | — | — | — | — | — | — | — | — | — | — | — | 9·6 | 1·4 | 8·2 | 4·2 |
| 12 | 3·7 | *0·2* | 3·5 | 4·0 | 2·3 | 0·3 | 2·0 | 5·4 | 6·2 | 3·8 | 2·4 | 4·6 | — | — | — | — |
| 14 | — | — | — | — | — | — | — | — | — | — | — | — | 9·3 | 1·4 | 7·9 | 5·0 |
| 16 | 2·5 | 0·7 | 1·8 | 4·6 | 7·2 | 1·6 | 5·6 | 5·2 | 4·8 | 0·5 | 4·3 | 5·0 | — | — | — | — |
| 18 | — | — | — | — | — | — | — | — | — | — | — | — | — | — | — | — |
| 21 | 3·8 | 0·6 | 3·2 | 4·2 | 5·9 | 0·9 | 5·0 | 4·0 | 6·8 | 1·4 | 5·4 | 5·2 | — | — | — | — |
| 29 | — | — | — | — | — | — | — | — | — | — | — | — | — | — | — | — |
| 50 | — | — | — | — | — | — | — | — | — | — | — | — | — | — | — | — |
| 56 | — | — | — | — | — | — | — | — | — | — | — | — | 2·8 | — | — | 5·6 |
| | | | | | | | | | | | | | | Exitus | | |
| 74 | — | — | — | — | — | — | — | — | 4·2 | — | — | 5·2 | | | | |
| | | | | | | | | | | Exitus | | | | | | |

| | 1000 oral | | | | 2000 oral | | | | 3000 oral | | | | 5 × 200 i.v. | | | |
|---|---|---|---|---|---|---|---|---|---|---|---|---|---|---|---|---|
| Days | W.B.C. $10^3$ | N $10^3$ | L $10^3$ | R.B.C. $10^6$ | W.B.C. $10^3$ | N $10^3$ | L $10^3$ | R.B.C. $10^6$ | W.B.C. $10^3$ | N $10^3$ | L $10^3$ | R.B.C. $10^6$ | W.B.C. $10^3$ | N $10^3$ | L $10^3$ | R.B.C $10^6$ |
| Before treatment | *9·6* | 6·7 | 2·9 | 5·2 | *12·0* | 4·8 | 7·2 | 5·2 | *5·7* | 0·8 | 4·9 | 5·6 | *6·8* | *2·4* | 4·4 | 5·2 |
| 1 | 10·0 | 7·7 | 2·3 | 5·6 | 9·9 | 4·8 | 5·1 | 5·6 | 5·5 | 2·2 | 3·3 | — | 2·1 | 0·8 | 1·3 | — |
| 2 | 7·7 | 6·4 | 1·3 | 5·0 | — | — | — | — | 4·0 | 1·8 | 2·2 | 5·2 | 2·1 | 0·5 | 1·6 | — |
| 3 | 4·2 | 1·5 | 2·7 | 4·2 | — | — | — | — | *2·1* | *0·01* | 2·09 | 4·8 | 2·3 | 0·5 | 1·8 | — |
| 4 | *4·2* | *1·1* | 3·1 | — | *2·8* | *0·05* | 2·75 | 5·0 | — | — | — | — | — | — | — | — |
| 5 | — | — | — | — | — | — | — | — | | Exitus | | | 0·9 | 0·1 | 0·8 | 5·2 |
| 6 | — | — | — | — | — | — | — | — | — | — | — | — | *1·7* | *0·2* | 1·5 | 4·8 |
| 7 | 11·0 | 3·5 | 7·5 | — | 11·3 | 5·65 | 5·65 | 4·6 | — | — | — | — | 4·8 | 2·3 | 2·5 | 4·2 |
| 9 | — | — | — | — | — | — | — | — | — | — | — | — | — | — | — | — |
| 11 | 12·7 | 8·9 | 3·8 | 4·4 | 13·3 | 7·3 | 6·0 | 5·2 | — | — | — | — | — | — | — | — |
| 12 | — | — | — | — | — | — | — | — | — | — | — | — | — | — | — | — |
| 14 | 14·4 | 10·8 | 3·6 | — | 9·5 | 4·75 | 4·75 | 5·0 | — | — | — | — | — | — | — | — |
| 16 | — | — | — | — | — | — | — | — | — | — | — | — | — | — | — | — |
| 18 | 10·7 | 8·3 | 2·4 | — | — | — | — | — | — | — | — | — | — | — | — | — |
| 21 | — | — | — | — | — | — | — | — | — | — | — | — | — | — | — | — |
| 29 | — | — | — | — | | Exitus | | | — | — | — | — | — | — | — | — |
| 50 | 18·0 | — | — | 8·6 | — | — | — | — | — | — | — | — | — | — | — | — |
| | | Exitus | | | | | | | | | | | | | | |
| 56 | — | — | — | — | — | — | — | — | — | — | — | — | — | — | — | — |
| 74 | — | — | — | — | — | — | — | — | — | — | — | — | — | — | — | — |
| | — | — | — | — | — | — | — | — | — | — | — | — | — | — | — | — |

for them to decrease in number a dose was required which was capable of reducing the number of neutrophils by more than 50 per cent ; even then, a relative lymphocytosis sometimes developed owing to the high degree of neutropenia. The thrombocyte count remained the same unless the drug was introduced in a quantity approaching the median lethal dose. Erythropoiesis was left intact even by that dose, but a lethal dose gave rise to haematologic changes resembling grave agranulocytosis, and red blood corpuscles began to be destroyed upon its action.

When the doses were administered in fractions, the changes in the blood picture were much more conspicuous.

In the bone marrow, smaller doses induced no particularly remarkable lesions in the mouse and the rabbit, although the bone marrow of the latter was slightly more sensitive. Doses of the drug approaching the maximally tolerated level produced significant reductions in the number of myeloid elements, but left the erythropoietic cells, the megakaryocytes and the reticular elements undamaged. Quantities in excess of the maximum tolerated dose gave rise to grave changes in the bone marrow, causing pycnotic disintegration of large numbers of cells and reducing the number of cells of intact structure ; the capillaries among them appeared dilated, and the remaining megakaryocytes become all the more conspicuous. Treatment even with large doses given for a long period of time failed to reveal changes pointing to serious lesions in erythropoiesis.

The tumour-inhibiting action of the drug is apparently selective : it differed, in fair degree in the six tumour types involved in our tests (Yoshida rat sarcoma, Guérin rat carcinoma, Ehrlich adenocarcinoma, Crocker sarcoma 180 in mice and the 36th passage of ENG spontaneous sarcoma in mice) (Table III). This obviously makes it desirable to test the compound against a wider spectrum of tumours for its growth-inhibiting activity.

TABLE III.—*Growth Inhibiting Action of MM on Transplanted Tumours*

| Type of tumours | Number of animals | Dose (mg./kg./day) | Percentage inhibition |
|---|---|---|---|
| Yoshida solid | 5 | 5 × 500 i.v. Killed on 6th day | 77 |
| Guérin | 10 | 9 × 500 i.v. Killed on 10th day | 95 |
| Crocker | 10 | 10 × 500 i.p. Killed on 11th day | 52 |
| ENG mouse-sarcoma | 10 | 5 × 500 i.p. Killed on 6th day | 87 |
| Ehrlich ascites | 10 | 6 × 200 i.p. Killed on 8th day | 36 |
| Ehrlich subcutaneous | 10 | 6 × 500 i.p. Killed on 8th day | 30 |

Generally speaking, MM has a more marked inhibitory effect on transplanted tumours of the rat than on those of the mouse. A fact meriting special attention is that on the Guérin tumour, known to be fairly resistant to most drugs of therapeutic effect, as much as 90 per cent inhibition of growth was seen, and on the intravenous administration of a very large quantity (9–10 × 500 mg./kg.), even recovery was attained. A much smaller amount of the drug sufficed to bring about recovery in Yoshida tumour.

The question of how MM affects survival has not yet been fully investigated.

Nevertheless, on the strength of experience that has been gained so far, it seems safe to say that, given in suitable doses, the compound is capable not only of retarding growth, but also of protracting survival time.

In tissue culture, MM was observed to have growth-inhibitory and destructive effects on fibroblasts of chick-embryo hearts and fresh Crocker sarcoma and HeLa cells. Cultures incubated for 24 hours were treated with solutions of various concentrations of the drug, and after an additional 24 hours the extent of damage was evaluated, and expressed by ±, +, ++, or +++. After adding sodium bicarbonate, to bring the pH from acid to neutral, a 1000 $\gamma$/ml. dose was enough to elicit a very marked effect, while double this dose was found to destroy all the cultures. With a solution which had not been treated to prevent a fall in pH (pH 5·8), a dose of as much as 5000 $\gamma$/ml. was required for the drug to exercise its maximum effect (Table IV). It is remarkable how nearly the doses found efficaceous in explants, agree with those established in our experiments *in vivo*. This seems to indicate that explantation might be of value in determining the doses.

TABLE IV.—*Effect of MM on Tissue Culture*

| | Fibroblast | | Crocker | | HeLa | |
|---|---|---|---|---|---|---|
| Gamma/ml. | 1 | 2 | 1 | 2 | 1 | 2 |
| 250 | .. | + | .. | + | .. | .. |
| 500 | + | + | + | + | + | .. |
| 1000 | +++ | + | +++ | +(+) | ++ | .. |
| 2000 | +++ | .. | +++ | .. | +++ | .. |
| 5000 | .. | ++(+) | .. | +++ | .. | .. |

1. pH : 7·0.
2. pH : 5·8.

In Ehrlich ascites tumour, MM—like chemotherapeutic agents in general—produced a reduction in the cell count, an increase in the number of eosinophils, and caused changes in cellular morphology and acridine-orange uptake. It is interesting to note that whereas after the introduction of most agents (chloroethylamino derivatives, antimetabolites, etc.) acridine-orange uptake decreases, it invariably increased upon the action of MM, quite like upon that of Myleran (Table V).

TABLE V.—*Changes in Number and Staining Capacity of Ehrlich Ascites Cells Following Treatment of MM*

| | Controls (%) | MM 500 mg./kg. intraperitoneal — Hours: 6 | 24 | 48 | 72 | 96 |
|---|---|---|---|---|---|---|
| Number of cells* | 100 | 51 | 36 | 39 | 30 | 16 |
| Cells staining with eosin† | 5 | 8 | 16 | 37 | 29 | 40 |
| Acridin orange-uptake‡ | 100 | 89 | 223 | 273 | 219 | — |

* $10^5$/ml. = 100 per cent.

† In percentage of total cells.

‡ $10^{-8}$ gamma/cell = 100 per cent.

Similarly to Degranol and several other chemotherapeutic drugs, MM prevents many of the intravenously introduced tumour cells from taking, and inhibits metastasis production. Twenty-four hours after the intravenous introduction of ten million Guérin carcinoma cells, each of a group of five animals was given into the veins seven consecutive daily doses of 500 mg. of MM, and another group was left untreated to serve as controls. All were sacrificed on the 24th day following transplantation. Autopsy revealed extensive metastases in the lungs, adrenals, and lymph nodes of the controls, but none in four of the treated experimentals, while in the fifth, only a few metastases were found, restricted to lymph nodes (Fig. 2). We regard this protective action of MM as significant since on the evidence of our earlier findings (Lapis and Németh, 1956) Myleran does not possess it ; in addition, it may prove to be of value before and after surgical treatment of tumours.

FIG. 2.—Effect of MM on the metastasis of intravenously induced Guérin carcinoma as observed on the 20th day.

In our studies of the dynamics of the morphological changes caused by MM, Guérin tumours were chosen : first, because the cells in their viable parts are fairly uniform ; secondly, because in Guérin tumours of roughly equal size, the proportion of the area of the viable parts to that of the circumscribed necrotic parts of moderate extent, is largely the same ; and thirdly, because MM powerfully inhibits the growth of this type of tumour. About ten days after transplantation, animals bearing tumours the size of a small walnut were selected and given intravenously a single 2000 mg./kg. dose of MM. These were killed one by one, 1, 3, 6, 10, and 12 hours, 1, 2, 3, 4, and 5 days, after injection. Sections were prepared of the tumours at the largest diameter, and of the more important organs. The morphological changes seen were essentially the same as those produced by the other alkylating agents (nitrogen mustard, Degranol, Sarcolysin, Mitomen, etc.). We describe them in the following (Table VI).

TABLE VI.—*Cytological Changes Upon the Action of MM*

| | Control | Hours after administration 10 | 24 | 48 | 72 | 92 | 120 |
|---|---|---|---|---|---|---|---|
| *Found in tumour* | | | | | | | |
| Intact cells | 6·7 | 0·8 | 0·2 | 0·1 | — | — | — |
| Mitosis, anomalous or disintegrating | 1·2 | 7·5 | 15 | 26·7 | 22·4 | 6·4 | 9·6 |
| Bi- and multinuclear cells | 3·6 | 3·8 | 17·7 | 40·4 | 78·6 | 47·5 | 49·3 |
| Necrosis | +++ | ++ | ++ | ++ | +++ | ++++ | ++++ |
| *Assayed in duodenum* | | | | | | | |
| Decrease in number of mitotic cells | .. | ++ | +++ | +++ | +++ | ++ | — |
| Anomalous mitotic cells | + | +++ | — | — | — | +++ | + |
| Disintegrating mitotic cells | — | ++ | +++ | ++ | + | — | — |
| Resting cells | + | + | + | ++ | +++ | +++ | ++ |
| Giant cells | .. | .. | .. | .. | + | + | .. |

\+ From sporadic to few.
++ From few to about half.
+++ From half to many.
++++ Between many and almost all.

After 12 hours, only cells in the process of mitosis are affected : the chromatin is loosened in structure or reduced to powder, the chromosomes have drifted away and broken into fragments, a bridge is formed between the two mitotic spindles, etc. After one day, but more conspicuously so after two days, anomalous mitoses increase in number, pycnotic nuclear destruction sets in, and binuclear and multinuclear cells suddenly appear. Thereafter, anomalous mitoses decrease in number, and pycnotic disintegration becomes so extensive within the viable parts as to loosen the structure of the tumour. The central necrotic areas keep growing in size until on the 4th or 5th day the entire tumour is but a necrotic mass, with only a very thin envelope of retained tumour tissue around it, in which most of the tumour cells, showing two or more nuclei are seen surrounded by an increasing number of connective tissue fibres (Fig. 3 and 4).

In the duodenum, mitoses were no longer seen after 10 hours, but pycnotic nuclei were encountered in very large numbers. By the third day, the villi were broadened and flattened, their connective tissue was extensively infiltrated by globular cells, there was cystic dilatation of the glands, and the epithelial cells were flattened or turned into multinuclear giant cells. By the fifth day, hardly any traces of these changes were seen (Fig. 5).

Morphological changes of the above description—enlargement of the necrotic areas, mitotic anomalies, and pycnotic disintegration of tumour cells—are seen in Crocker 180 mouse carcinoma and ENG mouse sarcoma, but they are of a much milder nature and require considerably larger doses to arise (3 to 6000 mg./kg.).

### SUMMARY

The compound prepared by linking the sulphonoxyd radical with mannitol is readily soluble in water, much less toxic than Myleran, and possesses superior biological qualities.

In tissue culture, on the use of a neutral medium it exercises a powerful destructive effect on fibroblasts, and Crocker and HeLa cells.

It is capable of potentially damaging Ehrlich ascites cells, as is evidenced by a decrease in their number upon its action, and an increase in their capacity to stain with eosin and acridine orange.

One tenth of the maximally tolerated dose of the compound suffices to give rise to marked changes in the blood picture ; the granulocytes in the blood stream and the myeloid elements of the bone marrow suffer most. Lymphocytes are left unaffected by doses of less than at least a 50 per cent neutrophil-depressing activity. Toxic doses are required to do damage to thrombocytes. The least effect the compound has is that on the erythropoietic system.

In six different types of rat and mouse tumours its growth-inhibiting action was found to range from 30 to 90 per cent. Recoveries were attained with it in Yoshida and Guérin tumours. Unlike Myleran, it inhibited metastatic diffusion to the organs after intravenous transplantation.

Its destructive effect on tumour cells, studied chiefly in Guérin rat carcinoma, is similar to but more lasting than that of the other alkylating agents.

Preliminary data obtained in as yet incomplete investigations indicate that the compound may possess a cumulative effect and a carcinogenic action which manifests itself early (mouse leukaemias, endotheliomas associated with ascites, etc.).

Our thanks are due to Dr. Martha Schmidt and Dr. Ibolya Fogarassy for divers laboratory tests carried out with rabbit blood ; to Dr. J. Sugár and Dr. Éva Gáti for investigations into the straining capacity of ascites tumours ; to I. Pályi and Emily Gréczy for tissue culture experiments ; and to G. Gálffy for his assistance in our toxicological and biological studies.

### REFERENCES

HADDOW, A., TIMMIS, G. M. AND BROWN, S. S.—(1958) *Nature*, **182**, 1164.
KELLNER, B. AND NÉMETH, L.—(1956) *Z. Krebsforsch.*, **61**, 165.
LAPIS, K. AND NÉMETH, L.—(1956) *Brit. J. Cancer*, **10**, 719.
VARGHA, L.—(1955) *Naturwissenschaften*, **42**, 582.
*Idem* AND KUSZMANN, J.—(1959) *Ibid.*, **2**, 84.
*Idem*, TOLDY, L., FEHÉR, Ö. AND LENDVAI, S.—(1957) *J. chem. Soc.*, 805.

---

### EXPLANATION OF PLATES

FIG. 3.—Morphological changes in Guérin rat carcinoma due to the action of MM :

(*a*) Controls. Viable portion. × 200.

(*b*) Picture seen 60 hours after injection of a single dose of 2000 mg./kg. dose. Note extensive pycnotic nuclear disintegration, loosened structure, and the presence of cells with two and more nuclei. × 200.

(*c*) Picture seen 120 hours after injection of the same dose. Note that most of the cells are now bi- or multinuclear and that there are now more connective tissue fibres. × 200.

FIG. 4.—(*a*) Great number of anomalous mitotic forms and disintegrating cells. Chromatin in resting cells clumped or of a lytic light colour. (Identical with Fig. 3*b* but × 1620.)

(*b*) Giant cells and increased amount of connective tissue. (Identical with Fig. 3c but × 1620.)

FIG. 5.—Pictures of the duodenum seen after administration of a single dose of 2000 mg./kg.

(*a*) Ten hours after injection : mitoses no longer visible ; numerous pycnotic nuclei and chromatin globules formed from nuclear fragments ; most cells at rest turned light in colour ; great number of goblet cells. × 220.

(*b*) Seventy-two hours after injection : duodenal mucous membrane very thin ; villi broadened ; glands dilated cyst like ; epithelial lining flattened ; infiltration of globular cells in connective tissue extending as far as the submucosa.